# Application of a Rational Crystal Contact Engineering Strategy on a Poly(ethylene terephthalate)-Degrading Cutinase

**DOI:** 10.3390/bioengineering12060561

**Published:** 2025-05-23

**Authors:** Brigitte Walla, Anna-Maria Dietrich, Edwin Brames, Daniel Bischoff, Stefanie Fritzsche, Kathrin Castiglione, Robert Janowski, Dierk Niessing, Dirk Weuster-Botz

**Affiliations:** 1 Biochemical Engineering, Department of Energy and Process Engineering, TUM School of Engineering and Design, Technical University of Munich, Boltzmannstraße 15, 85748 Garching, Germany; brigitte.walla@tum.de (B.W.); daniel.bischoff@tum.de (D.B.); 2 Institute of Bioprocess Engineering, Department of Chemical and Biological Engineering, Friedrich-Alexander-Universität Erlangen-Nürnberg, Paul-Gordan-Straße 3, 91052 Erlangen, Germany; stefanie.fritzsche@fau.de (S.F.); kathrin.castiglione@fau.de (K.C.); 3 Molecular Targets and Therapeutics Center, Institute of Structural Biology, Helmholtz Zentrum München, Ingolstädter Landstraße 1, 85764 Neuherberg, Germany; robert.janowski@helmholtz-muenchen.de (R.J.); niessing@helmholtz-muenchen.de (D.N.); 4 Institute of Pharmaceutical Biotechnology, Ulm University, James-Franck-Ring N27, 89081 Ulm, Germany

**Keywords:** protein engineering, crystallization, PET-degrading cutinase, downstream processing

## Abstract

Industrial biotechnology offers a potential ecological solution for PET recycling under relatively mild reaction conditions via enzymatic degradation, particularly using the leaf branch compost cutinase (LCC) quadruple mutant ICCG. To improve the efficient downstream processing of this biocatalyst after heterologous gene expression with a suitable production host, protein crystallization can serve as an effective purification/capture step. Enhancing protein crystallization was achieved in recent studies by introducing electrostatic (and aromatic) interactions in two homologous alcohol dehydrogenases (*Lb*/*Lk*ADH) and an ene reductase (*Nsp*ER1-L1,5) produced with *Escherichia coli*. In this study, ICCG, which is difficult to crystallize, was utilized for the application of crystal contact engineering strategies, resulting in ICCG mutant L50Y (ICCGY). Previously focused on the Lys-Glu interaction for the introduction of electrostatic interactions at crystal contacts, the applicability of the engineering strategy was extended here to an Arg-Glu interaction to increase crystallizability, as shown for ICCGY T110E. Furthermore, the rationale of the engineering approach is demonstrated by introducing Lys and Glu at non-crystal contacts or sites without potential interaction partners as negative controls. These resulting mutants crystallized comparably but not superior to the wild-type protein. As demonstrated by this study, crystal contact engineering emerges as a promising approach for rationally enhancing protein crystallization. This advancement could significantly streamline biotechnological downstream processing, offering a more efficient pathway for research and industry.

## 1. Introduction

Plastics and plastic packaging are integral to the global economy [1]. However, of the estimated 413.8 million tons of plastic produced worldwide in 2023, only about 8.7% were mechanically recycled, while alternative recycling processes, such as chemical recycling (0.1%), returned even smaller amounts of material to the circular economy [2]. For one of the major synthetic, petroleum-based polymers, poly(ethylene terephthalate) (PET), which is popular for its use in textiles (fibers) and packaging (foils and bottles) [1,3,4], the main recycling process, thermomechanical degradation, impairs its mechanical properties [5].

Biotechnology offers a potential ecological solution for PET recycling under relatively mild reaction conditions via enzymatic degradation to its building blocks, terephthalic acid and ethylene glycol. PET ester bonds can be hydrolyzed by subgroups of (serin) hydrolases, namely lipases, carboxylesterases, and cutinases [6,7,8,9]. In the past 25 years, studies have extensively characterized the applicability of these enzymes in PET degradation (reviewed in [4,6,8,10,11,12,13]).

Primarily, cutinases hydrolyze hydrophobic substrates in solution or emulsions [8,14], which has sparked interest in the role of cutinases in biotechnological PET degradation, particularly the leaf branch compost cutinase (LCC; PDB ID: 4EB0) [14,15,16]. Although LCC exhibits high thermodynamic stability (T_m_ = 86 °C), its kinetic stability is relatively low (dynamic t_1/2_ = 40 min at 70 °C, dynamic t_1/2_ = 7 min at 80 °C; [7,14]), while the protein is also prone to aggregation [16]. The need for high catalytic turnover and stability at temperatures above PET’s glass transition state during enzymatic hydrolysis (T_g_ > 65 °C; [9]) is crucial for the commercial viability of biocatalysts as chain mobility in the amorphous phase of PET improves access to ester bonds [17,18]. Computer-aided enzyme engineering performed by Tournier et al. [15] introduced an Ile at LCC position F243 to enhance the enzyme’s catalytic activity by further expanding the substrate-binding site. Additionally, thermostability was increased to 94 °C by disulfide bond formation between D238C and S283C in combination with mutation Y127G. Thus, the LCC quadruple mutant ICCG was engineered (PDB ID: 6THT; from here on named “ICCG”), being outstanding in its improved PET depolymerization productivity (16.7 g·L^−1^·h^−1^ terephthalic acid at 2 mg_enzyme_ g_PET_^−1^) [15].

Although the enzymatic processing of PET waste could provide a sustainable alternative to current methods for PET recycling, a significant bottleneck exists in industrial protein downstream processing: the protein purification/capture step. As conventional packed-bed chromatography is a cost-intensive and time-consuming bioseparation method [19,20,21], interest in the “anything but chromatography” approach has increased in recent years (reviewed in [22,23]). Along with high-throughput (HTP) process development, aqueous two-phase systems, and membranes and monoliths, Roque et al. [22] highlighted batch crystallization as an alternative to chromatography with the highest “technology readiness level” (the system is proven in an operational environment), as demonstrated in various studies [21,23,24,25]. In addition, crystalline products exhibit high purity and stability, positively impacting formulation, shelf life, storage costs, and drug delivery [21,26].

Despite these advantages, the main barrier to industrial adoption remains the complexity of biomolecules impacting the crystallization process, rendering the determination of suitable crystallization conditions non-transferable between protein families and thus empirical [23,27]. Enormous progress has been made in three-dimensional (3D) structure prediction using high-accuracy models [28], even for biological complexes (AlphaFold3; [29]). Nevertheless, AlphaFold was reported to show poor predictability regarding structural changes in single amino acid exchanges [30]. Also, defining crystal contacts for specific crystallization conditions remains empirical and challenging.

Recent studies addressed the challenge of crystallizability by intrinsically exchanging amino acid residues on crystal contacts, applying rational crystal contact engineering strategies [31,32,33]. Here, Grob et al. [32] defined “enhanced crystallizability” for the µL batch crystallization of *Lactobacillus brevis* alcohol dehydrogenase (*Lb*ADH) by using (i) a reduced crystallization induction time, (ii) an increased amount of crystals at the crystallization equilibrium equivalent to a higher nucleation rate, (iii) a reduction in crystal growth duration, (iv) a faster setting of crystallization equilibrium, and (v) an extended nucleation window towards lower crystallization agent and protein concentrations.

The *Lb*ADH, heterologously produced with *Escherichia coli*, was utilized to reproducibly apply rational crystal contact engineering strategies and to investigate the impact on crystallizability by single- and double-amino-acid exchanges. To enhance crystallizability, surface entropy reduction (exchanging long, flexible side chains (Arg and Lys) for shorter ones (Ala and Val)) [34,35,36] was applied (K32A), along with the introduction of aromatic interactions (Tyr and Phe) at a symmetrical crystal contact (D54F/Y) [32]. Introducing charged amino acid residues (Glu, His, and Lys) at crystal contacts increased the crystallizability of *Lb*ADH mutants T102E [32] and Q126H [31] by enhancing intermolecular electrostatic interactions. This engineering strategy was successfully transferred to the homologous *Lactobacillus kefiri* ADH (T102E and Q126K) [33], and was recently successfully applied to a non-ADH-homologous *Nostoc* sp. PCC 1720 ene reductase (e.g., *Nsp*ER1-L1,5 mutants Q204K and T354K) [37].

To further assess the generalization of the examined crystal contact engineering approaches, knowledge from previous studies [31,32,33] will be applied and expanded to the cutinase ICCG within this study. Recent studies [31,32,33,37] mainly focused on increasing crystallizability through amino acid exchanges at crystal contact sites, especially by exchanging Thr (T) with Glu (E) and Gln (Q) with Lys (K). Within the scope of this study, the approach of introducing electrostatic interactions should be further broadened to Glu-Arg interactions. In addition, the rationale of the crystal contact engineering approach should be further examined by performing amino acid exchanges (T→E and Q→K) not only on crystal contacts but also as negative controls on non-crystal contact sites. Following the established strategy, no increase in crystallizability should be observed (maintaining the same crystal system). The combined crystal contact engineering results on *Lb*ADH, *Lk*ADH, and *Nsp*ER1-L1,5, augmented with the results of this study on ICCG, should support the assumption of a generalized approach. Furthermore, it should demonstrate the potential of crystal contact engineering to improve protein crystallization, which can contribute to more efficient biotechnological downstream processing.

## 2. Materials and Methods

Chemicals were purchased from Carl Roth (Karlsruhe, Germany), except for PEG 6000 (Merck, Darmstadt, Germany) and p-nitrophenyl acetate (pNPA, Thermo Fisher Scientific, Schwerte, Germany). Biomaterials (enzymes, protein markers, and kits) were purchased from New England Biolabs (NEB, Ipswich, MA, USA).

### 2.1. Site-Directed Mutagenesis

The leaf branch compost cutinase (LCC) quadruple mutant ICCG (PDB ID: 6THT) was selected for the application of the crystal contact engineering strategy. Site-directed mutagenesis of the *iccg* gene (encoded on pET26b; plasmid received from the Institute of Bioprocess Engineering, FAU, Germany; [38]) was performed using a standard QuikChange PCR protocol with adaptations in primer design according to Zheng et al. [39], with partial overlapping oligonucleotides used for the ICCG mutants, as listed in Supplementary Table S1. Plasmid amplification and verification of the correct mutants’ open reading frame were performed according to Walla et al. [33]. As Fritzsche et al. [38] removed the 34-amino-acid-long N-terminal pelB signal sequence of LCC (PDB ID: 4EB0), resulting in intracellular protein maturation, the mutation positions in this study follow the sequence numbering of Fritzsche et al. [38].

### 2.2. Heterologous Protein Production

For recombinant protein production on an mL scale, 100 µL of chemically competent [40] *E. coli* strain BL21 (DE3) cells were transformed with approx. 50 ng of purified pET26b-ICCG DNA. Analogous to Nowotny et al. [31], individual colonies formed after transformation were transferred to preculture tubes filled with 6 mL terrific broth medium (TB; containing 35 mg L^−1^ kanamycin) and incubated overnight (max. 16 h, 37 °C, 180 rpm). For the main culture, shake flasks filled with 0.2 L TB medium (35 mg L^−1^ kanamycin) were inoculated with preculture (OD_600_ = 0.05) and incubated at 37 °C (200 rpm; Infors Multitron, Infors AG, Bottmingen, Switzerland) until OD_600_ = 0.7–1.0 was reached. Then, *iccg* gene overexpression was induced by adding 0.2 mM isopropyl β-D-1-thiogalactopyranoside (IPTG) while decreasing the temperature for protein production to 21 °C. After precisely 20 h of protein production, the cells were harvested (4 °C, 1500× *g*, 15 min; Rotixa 50 RS, Hettich, Tuttlingen, Germany), resuspended in phosphate-buffered saline (PBS, pH 7.4), and centrifuged again before the pellets were stored at −18 °C until further processing.

### 2.3. Protein Processing: Purification, Desalting, and Buffer Exchange

ICCG variants with C-terminal His_6_ tag were purified via two-step immobilized metal-affinity chromatography (IMAC), analogous to the method used by Fritzsche et al. [38]. The cell pellets (1–2 g) were resuspended in 12 mL IMAC binding buffer (20 mM Tris, 300 mM NaCl, 10 mM imidazole, pH 8.0), completed with non-specific protease inhibitor (1 mM phenylmethylsulphonyl fluoride, PMSF) and 10 mg L^−1^ DNase I. The cell suspension was sonicated (2 × 5 min, 55% intensity, 50% pulse; Sonopuls HD 2070, MS 73, BANDELIN electronic, Berlin, Germany) on ice, and the cell debris was subsequently removed by centrifugation (4 °C, 12,000× *g*, 1 h; Rotanta 460 R, Hettich, Tuttlingen, Germany). After loading the filtered (0.2 μm) supernatant onto an equilibrated HisTrap HP column (Cytiva, Chicago, IL, USA), the column was washed with 4 column volumes (CVs) of binding buffer, combined with 6% elution buffer (20 mM Tris, 300 mM NaCl, 500 mM Imidazole, pH 8.0), to remove non-specifically bound protein. The ICCG variants were eluted within 4 CVs and collected. The eluates’ high imidazole concentration was reduced immediately using PD-10 desalting columns packed with Sephadex G-25 resin (Cytiva, Chicago, IL, USA). According to the manufacturer’s gravity protocol, the ICCG variants were eluted with 3.5 mL storage buffer (300 mM NaCl, 20 mM Tris, pH 8.0). The purity of each ICCG variant (>95%) was verified by sodium dodecyl sulfate-polyacrylamide gel electrophoresis (SDS-PAGE).

As the IMAC-purified LCC ICCG wild type did not crystallize on a µL scale (batch crystallization), size exclusion chromatography (SEC) was subsequently performed to further purify the IMAC eluates. IMAC elution fractions were concentrated to 500 μL (Amicon centrifugal filter units, 10 kDa MWCO; Merck, Darmstadt, Germany) and loaded onto a Superdex 75 Increase 10/300 GL column (M_r_~3–70 kDa; Cytiva, Chicago, IL, USA). Fractions of the monodisperse peak were eluted using the storage buffer and used for HTP crystallization condition screening.

### 2.4. Static Protein Crystallization

The crystallization conditions for the ICCG wild type were determined via HTP screening using commercial plates: Index HT, Natrix, PEGRx HT (Hampton Research, Aliso Viejo, CA, USA), and JCSG+ (Jena Bioscience, Jena, Germany). Sitting drop crystallization was performed using vapor diffusion with a concentrated IMAC- and SEC-purified ICCG protein solution (10 g L^−1^; 300 mM NaCl, 20 mM Tris, pH 8.0). 

For the batch crystallization experiments, IMAC-purified, desalted, and concentrated ICCG variants (10 g L^−1^) were combined 1:1 with crystallization buffer containing 1.0–1.2 M succinic acid (pH 7.0), 0–0.1 M HEPES, and 0–50 g L^−1^ PEG 2000/6000. A 10 µL droplet was placed in a well of an MRC under an oil crystallization plate (SWISSCI, Neuheim, Switzerland), which was immediately sealed. Crystal growth was monitored automatically with a light microscope inside an incubator (20 °C), as described by Walla et al. [33]. Crystal length (in µm) was measured manually via NIS Elements AR imaging software (v. 5.02; Nikon, Düsseldorf, Germany). Statistically significant differences in crystal size were determined using a two-sided independent samples *t*-test (Microsoft Excel 365, v.2501) with different variances (significance level α = 0.05).

### 2.5. Protein Analytics: Electrophoresis and Esterase Assay

To determine the purity of ICCG variants in the IMAC eluate (0.5 g L^−1^) and to characterize the protein composition in the *E. coli* cell debris pellet and cell lysate, a discontinuous SDS-PAGE was performed according to the protocol of Laemmli et al. [41] ((bis-) acrylamide: 15% (*v*/*v*) in separating gel, 3% (*v*/*v*) in collecting gel; 35 mA per gel, 300 V, 1 h), and the focused protein bands were dyed according to Fairbanks et al. [42].

The enzymatic activity of the ICCG variants was determined spectrophotometrically according to Bhunia et al. [43]. The enzymatic activity assay was based on the enzymatic cleavage of the chromogenic substrate pNPA, resulting in the release of p-nitrophenol (pNP, λ_max_ = 405 nm), indicated by a color change from transparent to yellow. Analogous to the method used by Fritzsche et al. [38], 400 ng of IMAC-purified and dialyzed ICCG protein solution was combined with 130 μL buffer (10 mM NaCl, 20 mM Tris, pH 8.0), completed with 100 μM pNPA (prepared in acetonitrile), to a final volume of 250 µL. The release of pNP (ε = 17.4 mM^−1^ cm^−1^) was monitored in triplicate at λ = 405 nm (10 min, 3 s intervals, 37 °C) in a plate reader (Multiskan FC photometer, Thermo Fisher Scientific, Germany). The absolute enzymatic activity of the ICCG variants was calculated according to Fritzsche et al. [38], and the enzymatic activity of the mutants was evaluated in relation to the wild type (WT = 100%).

### 2.6. X-Ray Diffraction, Data Collection, Processing, and Refinement

X-ray diffraction and data collection, processing, and refinement were performed according to Walla et al. [37]. X-ray diffraction and data collection were performed at the DESY synchrotron (Hamburg, Germany; PETRA III, beamline 11). For data processing by molecular replacement with PHASER (v.2.8.3) [44], the crystal structure of ICCG (PDB ID: 6THT; [15]) was used as a search model. Subsequently, the model was refined using REFMAC (v.5.8) [45] and COOT (v.0.9.8) [46]. The resulting final model and structure factors were deposited in the Protein Data Bank (PDB) under identification codes (IDs) 9QYP (ICCG), 9QYU (ICCGY), 9QYQ (ICCGY T26E), 9QYT (ICCGY T110E), 9QYR (ICCGY Q183K), and 9QYS (ICCGY Q238K). Quality indicators for the X-ray diffraction datasets and refinement results are listed in Supplementary Table S2.

## 3. Results

**Figure 1:**
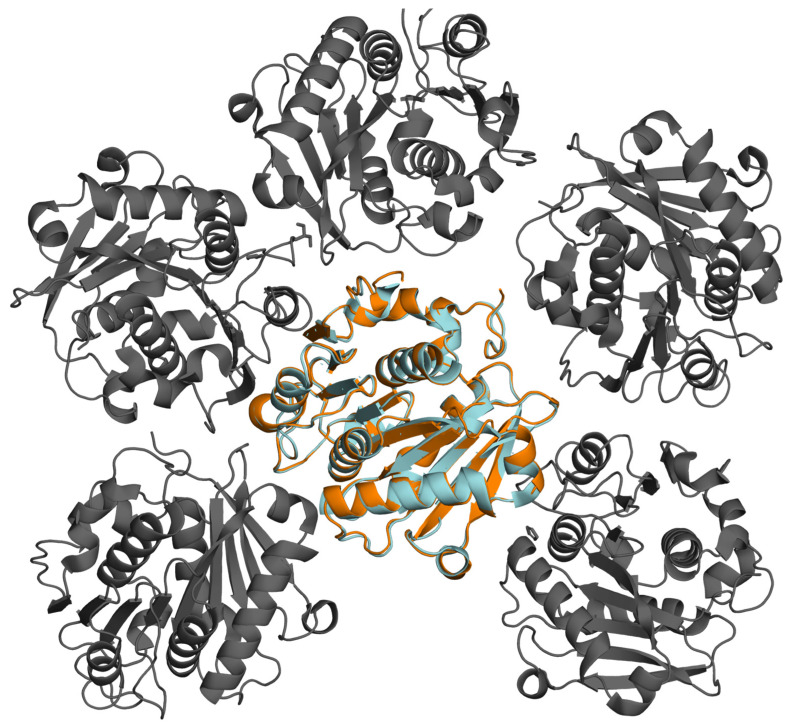
The alignment of an ICCG variant [38] structure (relaxed model generated with AI-based AlphaFold 2.0 [28], orange) to a published ICCG crystal structure (PDB ID: 6THT, cyan) with an RMSD of 0.248 Å, depicted in the crystal contact environment of the published structure (gray). The structures were aligned, and the illustration was generated using PyMOL (v.2.3, [47]).

Although 3D structure prediction models with high accuracy exist (AlphaFold [28,29]), the prediction of crystal contacts is—to date—not possible. Also, the predictability of structural changes due to amino acid exchanges using AlphaFold is low [30]. Thus, a high-resolution crystal structure is crucial for performing protein engineering specifically at crystal contacts. To overcome this prerequisite, this study approached engineering crystal contact interactions using a homology model. Here, a 3D structure for the N-terminally modified ICCG variant with a C-terminal His_6_ tag ([38]; see Section 2.1) was generated with AlphaFold [28] and aligned to the published LCC variant ICCG (PDB ID: 6THT [15]), as depicted in Figure 1 (root mean square deviation, RMSD = 0.248 Å).

Out of five mutated surface positions for introducing an electrostatic interaction (Glu-Lys/Arg), IMAC-purified ICCG mutant Y61E, generated from the crystal contact analysis of an ICCG homology model (6THT, Figure 1), was successfully crystallized in µL batch experiments with established HTP conditions (0.8 M succinic acid, pH 7.0, and 1.0 M succinic acid, 1% (*w*/*v*) PEG 2000, 0.1 M HEPES, pH 7.0). Mutant Y61E with the intended interaction between Y61E and R107 is depicted in silico in Supplementary Figure S1. Unfortunately, mutant Y61E revealed a loss of function. As the preservation of enzymatic activity is crucial for this study’s purpose of increasing crystallizability for technical crystallization, mutant Y61E was not considered further.

Simultaneously, the batch crystallization for ICCG was implemented as the crystallization results of generated ICCG mutants need to be compared to a wild type within the same experiment (as established for different proteins [32,33,37]) to assess the amino acid exchange impacting the crystallizability. Therefore, ICCG protein was heterologously produced with *E. coli* and purified via IMAC and SEC consecutively, as described in Section 2.2 and Section 2.3.

For batch crystallization experiments, the crystallization conditions of published ICCG PDB entries (ID: 4EB0, 6THT; Table 1) were tested first, with no crystals grown within two weeks. This duration was set as the maximum, as time is a crucial parameter for efficient technical crystallization (see the introductory definition of “enhanced crystallizability”).

Immediately afterward, the screening of crystallization conditions for the ICCG variant was extended to four commercial HTP screening plates using sitting-drop vapor diffusion crystallization (Section 2.4). Here, orthorhombic ICCG crystals grew in 2 out of 384 tested reservoir solutions within two weeks: 3 crystals (50–100 µm) grew in 0.8 M succinic acid (pH 7.0), and 12 crystals (50–100 µm) grew in 1.0 M succinic acid, 1% (*w*/*v*) PEG 2000, 0.1 M HEPES (pH 7.0). As for the sitting drop crystallization setup used in HTP screenings, the crystallization conditions in the droplet on the plate increased linearly during vapor diffusion. Thus, the crystal nucleation conditions can only be estimated. Transferring to batch crystallization with defined initial nucleation conditions is therefore challenging. Additionally, the cutinase ICCG is not easily crystallizable, as the success rate of the HTP screening process shows (2/384 conditions). As a result, crystallization was not reproducible for ICCG in batch experiments with the crystallization conditions obtained from HTP screening, tested using 0.4–1.8 M succinic acid and 0–5% PEG 2000 (2.5–10 g L^−1^ protein). 

**Figure 2:**
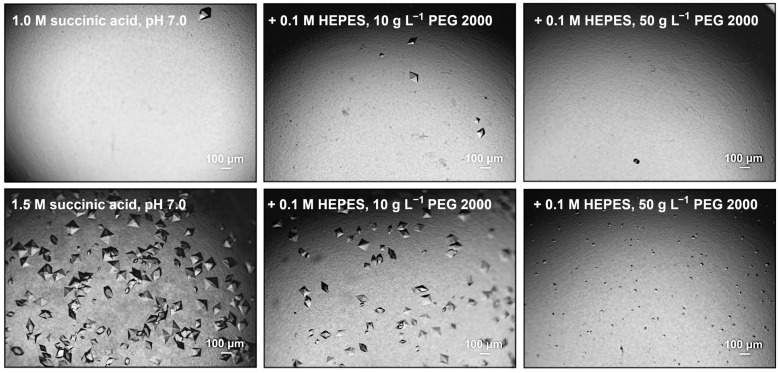
Batch crystallization of IMAC-purified ICCG mutant L50Y (ICCGY). Photomicrographs were taken after 56 h (20 °C) in crystallization buffers containing 1–1.5 M succinic acid at pH 7.0, 0–0.1 M HEPES, and 0–50 g L^−1^ PEG 2000 with protein concentration of 10 g L^−1^.

Thus, ICCG mutants were designed based on previous crystal contact engineering studies [32,33,37], with the ICCG structure obtained from vapor diffusion experiments (0.8 M succinic acid at pH 7.0), aiming to introduce electrostatic or aromatic π-π interactions, as described in the introduction. Out of seven mutated surface positions (L32, L50, S111, S194, A206, S207, and I209) for introducing an aromatic π-π interaction (Tyr, Trp, Phe), IMAC-purified ICCG mutant L50Y (named ICCGY subsequently), successfully crystallized in µL batch experiments with the established HTP conditions tested (0.8 M succinic acid at pH 7.0; 1.0 M succinic acid, 1% (*w*/*v*) PEG 2000, 0.1 M HEPES at pH 7.0). In addition to the successful implementation of batch crystallization for ICCGY, the nucleation window for this mutant is broadened to varying concentrations of succinic acid and PEG 2000, as depicted in Figure 2.

The experimental setup of the above-mentioned ICCG variants used to obtain protein crystals for X-ray analysis is summarized in Table 1.

When analyzing the crystallographic data of the ICCG variants, different space groups were determined for ICCG (I222, orthorhombic crystal system) and ICCGY (P4_1_2_1_2, tetragonal crystal system), also differing from the published PDB entry (6THT: P6_3_, hexagonal crystal system).

**Figure 3:**
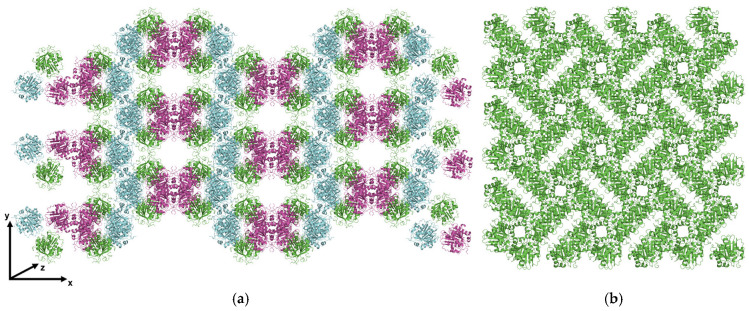

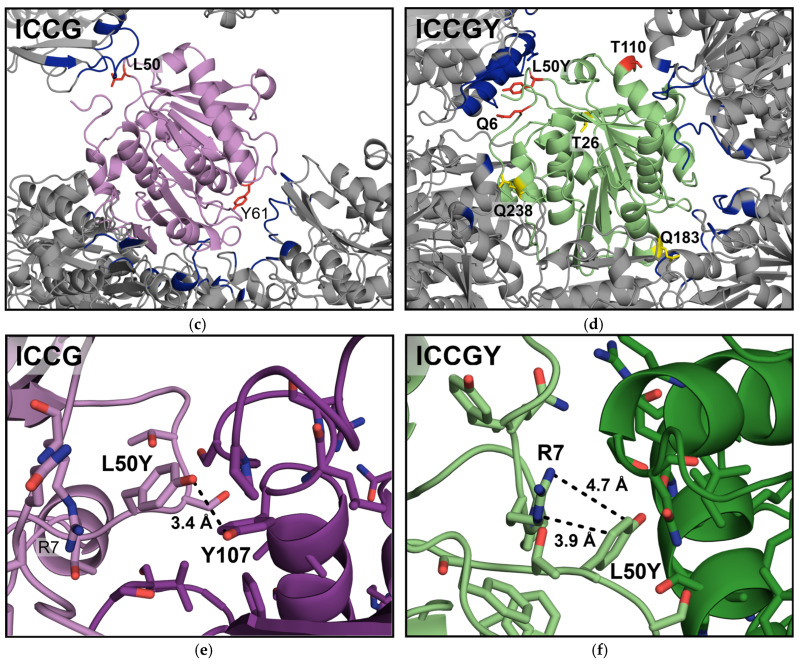
An illustration of the crystal packing of (**a**) ICCG (I222, PDB ID: 9QYP), retrieved from vapor diffusion experiments (0.8 M succinic acid pH 7.0), and (**b**) ICCG mutant L50Y (ICCGY; P4_1_2_1_2; PDB ID: 9QYU), retrieved from batch crystallization (1.2 M succinic acid at pH 7.0), with each color indicating one monomer of the asymmetrical unit. The crystal contact environment of a monomer is depicted (**c**) for ICCG and (**d**) for ICCGY. Crystal contacts (< 6 Å in distance from the central chain (purple/green)) are highlighted in blue, along with the tested crystal contact mutations (red) and negative controls (yellow). A section of an ICCG(Y) monomer focused on crystal contact position 50 for (**e**) in silico mutated L50Y (PDB ID: 9QYP) and (**f**) ICCGY (PDB ID: 9QYU), with differing neighboring amino acids at the crystal contact (neighboring monomers are colored in light/dark purple/green, respectively). The in silico mutant and figures were generated using PyMOL (v.2.3; [47]).

The change in the crystal system of ICCG(Y) from I222 to P4_1_2_1_2 is associated with a denser-packed ICCGY crystal structure, resulting in smaller crystal channels (Figure 3a,b). Also, the two space groups are formed by varying crystal contacts, resulting in different neighboring amino acids for L50 and L50Y (Figure 3c–f).

Although a π-π interaction between L50Y and Y107 was intended (Figure 3c,d), the analysis of the crystal structure of mutant ICCGY (Figure 3d) indicated an interaction with neighboring R7 (cation-π interaction). Nevertheless, ICCGY was used for crystal contact engineering studies to increase crystallizability, as this mutant crystallized reproducibly in batch crystallization experiments.

For the purpose of applying crystal contact engineering strategies, the amino acid distribution on the surface of ICCGY (total of 267 amino acids) was evaluated. Among the seven glutamines (Q6, 100, 143, 183, 190, 238, and 260), which are located on the ICCGY surface, Q183 is not located at a crystal contact (no neighboring intermolecular amino acid residue within the range of <10 Å). Q283, located at a crystal contact, shows no potential interacting amino acid (with negative charge/polar) in the range for an interaction (<10 Å). Furthermore, none of the four glutamic acids of ICCGY (E142, 174, 202, and 261) is in the range (<8 Å) to a Gln. Therefore, no Q→K mutants enhancing the crystallizability were designed. Instead, two negative controls with Q→K exchanges at non-crystal contacts and without an interacting amino acid in range were introduced, which should not increase crystallizability. The four endogenous lysines (K) at the protein’s surface are also not located at crystal contacts. Thus, not a Glu-Lys interaction (as intended in [32,33,37]), but a Glu-Arg interaction, was anticipated for the introduction of an electrostatic interaction at the crystal contact: Q6E (R139) and T110E (R119). The resulting ICCGY mutants are listed in Table 2, along with the applied strategy and potential interaction partner.

The results of the μL batch crystallization experiments for the IMAC-purified ICCGY wild type (WT) and mutants Q6E, T26E, T110E, Q183K, and Q238K are listed in Table 3. Differences in crystallization onset as a parameter for “increased crystallizability” compared to the WT, as well as trends in crystal size and amount, were analyzed.

**Figure 4:**
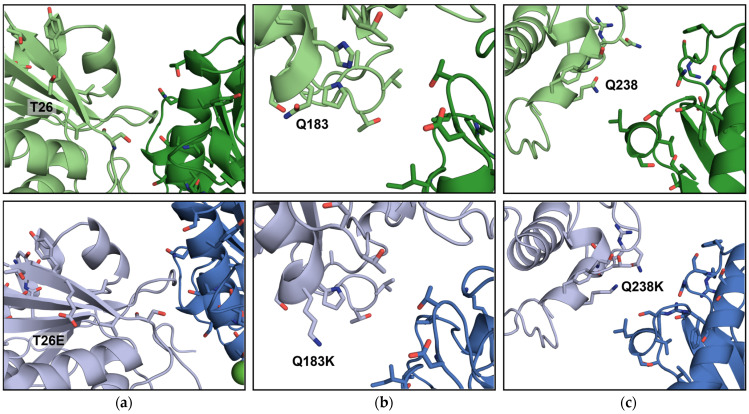
Illustration of ICCGY crystal contacts for wild type (**top**) and mutants (**bottom**) at positions (**a**) T26 (E), (**b**) Q183 (K), and (**c**) Q238 (K). The Cl^−^ ion is colored green. Figures were generated using PyMOL (version 2.3, [47]).

**Figure 5:**
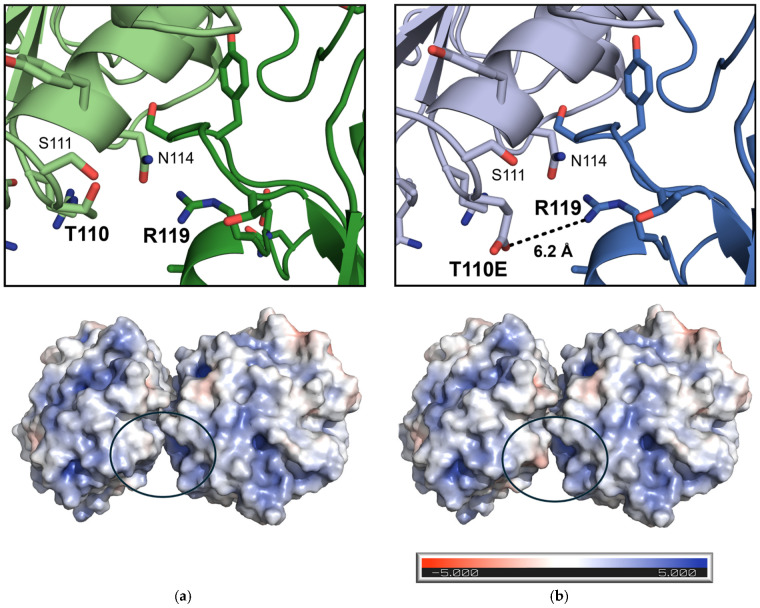
An illustration of ICCGY crystal contact at position 110 for the (**a**) wild type (green) and (**b**) mutant T110E (position circled in blue; neighboring monomers colored in light/dark green/blue, respectively). The surface net charge of two monomers from each ICCGY variant (generated via the “APBS electrostatics” plugin of PyMOL) is depicted below. The bar indicates the level of potential (in e) at the solvent-accessible surface from negative (red) to positive (blue). The net charge of ICCGY (pI = 9.17) is +5, resulting from 15 negatively (Glu and Asp) and 20 positively (Lys and Arg) charged amino acids. The figures were generated, and the distance was calculated using PyMOL (version 2.3 [47]).

ICCGY mutants Q6E and Q238K did not crystallize under the tested conditions within 56 h, rated as “inferior” to the ICCGY WT. Mutant T26E crystallization was microscopically visible after 22 h, similar to the WT’s crystallization onset under the same conditions (26 h). However, T26E crystallized in larger (344.5 µm ± 63.2) and fewer (12) crystals compared to the WT (247.8 µm ± 70.9; 25 crystals), indicating a trend to a reduction in nucleation events. Mutant Q183K also crystallized in a lower amount (12) with a mean crystal size similar to the WT (252.9 µm ± 97.2). The duration until the onset of crystallization was nearly doubled (48 h) compared to the WT, rendering it “inferior” to the WT. On the contrary, the occurrence of initial crystals for mutant T110E (6 h) was highly decreased compared to the WT, with the crystal size slightly increasing with a comparable crystal amount (305.7 µm ± 78.2; 21 crystals). Additionally, the mutant crystallized at a reduced protein concentration (5 g L^−1^), indicating a shift in the nucleation window to reduced crystallization conditions. The crystallization onset at a reduced protein concentration was also slightly earlier (20 h). To compare the experimental batch crystallization results on a molecular level, the crystal contacts of ICCGY mutants T26E, T110E, Q183K, and Q238K—all crystallizing in space group P4_1_2_1_2—are depicted in Figure 4 and Figure 5.

As shown in Figure 4, no interaction partner could be identified for the mutants T26E, Q183K, and Q238K within the standard range of electrostatic interactions (<4 Å), as intended for the selected negative controls. Furthermore, the crystal contacts of the three mutants remained comparable to the ICCGY WT.

In crystals with the mutant T110E protein, the distance to intermolecular R119 was reduced to 6.2 Å (Figure 5b). Although one T110E rotamer reduced the distance to R119 even more (4.5 Å), this rotamer clashed with neighboring N114. Nevertheless, negatively charged T110E, surrounded by an uncharged surface, pointing to the positive cavity of R119, supported the establishment of a long-range electrostatic interaction (Figure 5b).

**Figure 6:**
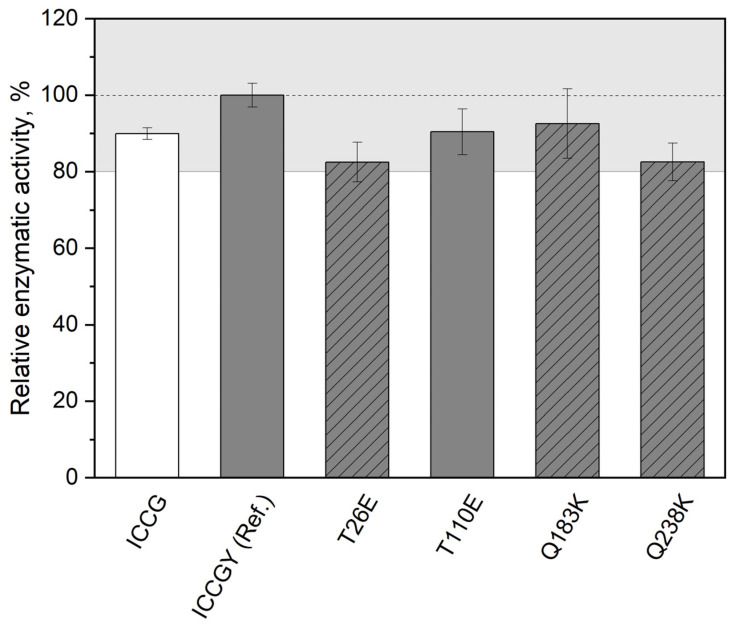
Maximum enzymatic activity of ICCG (white bar) and ICCGY mutants T26E, T110E, Q183K, and Q238K (gray bars, data for mutant Q6E not available), relative to ICCGY reference (Ref., set to 100%). IMAC-purified and dialyzed protein solutions of ICCG(Y) variants were provided (400 ng) as quintuplicates. Enzymatic activity was measured spectrophotometrically for 10 min at 405 nm (37 °C, every 6 s) with addition of 130 μL buffer (10 mM NaCl, 20 mM Tris, pH 8.0) containing 100 μM pNPA (final volume of 250 μL). “Negative control” ICCGY mutants are marked with stripes.

As it is crucial for biocatalysts to maintain activity, enzymatic activity was measured spectrophotometrically via an esterase assay (Section 2.5), and the results are depicted in Figure 6 with reference to ICCGY. The enzymatic activity of the tested ICCGY variants remained comparable to ICCG (within the range of ±20%).

## 4. Discussion

Enzymatic degradation of PET using suitable hydrolases, like the leaf branch compost cutinase (LCC) variant ICCG, offers potential for PET recycling under relatively mild reaction conditions. To improve the efficient downstream processing of this biocatalyst, protein crystallization can serve as an effective purification/capture step. In this study, ICCG was utilized for the application of a crystal contact engineering strategy to increase crystallizability and test the rationality of the applied approach. As focus has recently been placed on Lys-Glu interactions [36], the applicability of the engineering strategy was extended here to Arg as a further positively charged amino acid (Arg-Glu interactions). Furthermore, the rationale of the engineering approach is demonstrated by introducing Lys and Glu at non-crystal contacts or sites without potential interaction partners (“negative controls”).

Although the crystallization of LCC variant ICCG was per se possible in vapor diffusion experiments (PDB ID: 6THT, 7DS7), implementing batch crystallization for ICCG proved not applicable with the published crystallization conditions. The cutinase LCC is prone to aggregation [16], which is promoted at higher protein concentrations due to increased intermolecular hydrophobic or electrostatic surface interactions [48,49]. This renders the protein crystallization of LCC variants challenging, as a supersaturated state needs to be reached for nucleation [50]. The overall poor success rate of crystallization screenings [27] also applies to ICCG (0.5%). Nevertheless, this study managed to not only generate singular LCC ICCG crystals for crystallographic purposes but also to implement a reliable experimental setup for reproducibly crystallizing ICCG mutant L50Y (ICCGY) variants, which is essential for applications in downstream processing. For ICCG, two purification steps (IMAC and SEC) were required to crystallize in HTP screening, with no crystallization in subsequent batch crystallization experiments. However, the purity and homogeneity of ICCGY variants after one-step purification via IMAC were sufficient for HTP screening and successful batch crystallization, eliminating the need for SEC as a second purification step.

Molecular structure analysis revealed a change in the crystal system for ICCG (space group I222) and ICCGY (space group P4_1_2_1_2). Given the complexity of the crystallization process, with many factors affecting the crystal symmetry, the changing space group could result from the difference in protein solution homogeneity (see Supplementary Figure S2) and thus, potentially varying oligomeric states [51]. Additionally, the change in the crystallization method (see Table 1), influencing the external conditions [52], may have impacted the space group. The consistent space group among the crystallized variants of this study (ICCGY and mutants T26E, T110E, Q183K, and Q238K), all crystallized in batches with identical conditions, emphasizes these assumptions.

As the shift in the space group (I222 to P4_1_2_1_2) is linked with the formation of different crystal contacts, no π-π interaction between L50Y and Y107 was established at the ICCGY crystal contact, but presumably an intramolecular cation-π interaction (with R7, Figure 3). This result renders the availability of a crystal structure retrieved from the same crystallization conditions essential for rational crystal contact engineering [53] unless a reliable method for predicting crystal contacts for proteins across varying crystallization conditions is available. Advances in the prediction of contacts are being made with AI-based prediction tools—currently for biological complexes [29]. 

After successfully implementing a batch crystallization setup for ICCG variant L50Y, ICCGY mutants were generated according to strategies to either increase crystallizability (Q6E and T110E) or, as a control, not to increase crystallizability (negative controls T26E, Q183K, and Q238K). The crystallization results of four out of five mutations (except Q6E) complied with these strategies. The three negative control ICCGY mutants were meant to crystallize, but not with increased crystallizability compared to the WT, which could be demonstrated in this study (Table 3). Furthermore, mutations T26E, Q183K, and Q238K neither resulted in unintended electrostatic interactions nor affected crystal packing, as the crystal contacts remained similar (Figure 4). Thus, the rationale of the established crystal contact engineering strategy [32,33,37] of intentionally introducing electrostatic interactions to enhance protein crystallization could be further strengthened due to this new aspect.

Carugo et al. [54] reported the participation of protein N-/C-termini in crystallization due to the flexibility of the solvent-exposed termini during nucleation. Accordingly, alterations near the N-/C-termini could impact crystal packing interactions. As the ICCGY mutant Q6E is located near the N-terminus on a loop with no stable secondary structure, the interference of this mutation with the crystallization process is probable.

The introduced Glu-Arg interaction at the ICCGY T110E crystal contact represents a broader scope of application of the established crystal contact engineering strategy to introduce electrostatic interactions. As no suitable lysines were present at the ICCGY crystal contacts, the authors chose Arg as a positively charged substitute for the engineering strategy. The proximity of ICCGY mutant T110E to R119 (Figure 5) indicates an established long-range (5–10 Å; [55]) electrostatic Glu-Arg interaction at the crystal contact, confirming the successful extension of the approach. These molecular observations from X-ray structure analysis can be validated with experimental crystallization data. The nucleation window shift of T110E to a decreased protein concentration (5 g L^−1^) combined with a 77% reduction in the lag phase until crystallization (6 h) compared to the ICCGY WT (10 g L^−1^; 26 h) fulfill two criteria for increased crystallizability, as defined in the introduction. The ICCG mutant Y61E, generated in the first engineering attempts with a homology model, also established a Glu-Arg interaction (with R117; Supplementary Figure S1). This interaction is also known to be a more favorable crystal contact pairing than Tyr-Arg [56], emphasizing the extension of the crystal contact engineering strategy. Nevertheless, it is crucial for biocatalysts to maintain activity, so single amino acid exchanges at the protein surface were chosen at sites off the active site and, thus, should not interfere with enzymatic activity. All tested ICCGY variants showed comparable activity to ICCG (±20%).

## 5. Conclusions

In summary, this study successfully (i) applied the crystal contact engineering strategy to a new enzyme, the leaf branch compost cutinase variant ICCG; (ii) expanded the established crystal contact engineering strategy to Arg as a further amino acid for introducing electrostatic interactions; and (iii) implemented a reliable batch crystallization setup for the ICCG variant ICCGY. The negative controls T26E, Q183K, and Q238K, which did not aim to enhance interaction at the crystal contacts, showed no increase in crystallizability. In contrast, mutant T110E, which was designed to introduce an electrostatic interaction at the crystal contact, crystallized better than the ICCGY wild type regarding crystallization onset and had an extended nucleation window towards reduced protein concentrations. Future work may assess the differences between the WT and mutants in nucleation and crystallization kinetics via methods such as dynamic light scattering (DLS) or second-order nonlinear optical imaging of chiral crystals (SONICC) [57].

The two enhanced crystallization kinetic characteristics observed for the ICCGY mutant T110E, namely a shorter nucleation induction time and a shifted nucleation zone, significantly contribute to efficient downstream processing, enabling reduced protein concentrations and more time-efficient purification. Ultimately, this work contributes to the availability of ICCG as an efficient biocatalyst for ecological PET recycling.

## Figures and Tables

**Table 1:** A list of ICCG variants which were used as models for the experimental setup and for crystal contact engineering (PDB ID: 6THT), and the variants generated in this study (ICCG WT (PDB ID: 9QYP); ICCG L50Y (ICCGY, PDB ID: 9QYU)). The following chromatography methods were used for protein purification: Ion exchange (IEX), size exclusion (SEC), and immobilized metal ion affinity (IMAC). Also listed are the crystallization method, the crystallization conditions used for the ICCG variants, and the respective space group obtained after the X-ray analysis.

LCC Variant, (PDB ID)	Protein Purification	Crystallization Method	Crystallization Conditions	Space Group
ICCG (6THT; [15])	IEX, SEC	Vapor diffusion	0.1 M imidazole, 1 M sodium citrate, pH 8.0 (12 °C)	P6_3_
ICCG WT (9QYP)	IMAC, SEC	Vapor diffusion	0.8 M succinic acid pH 7.0 (20 °C)	I222
ICCG L50Y (9QYU)	IMAC	Batch crystallization	0.8 M succinic acid pH 7.0 (20 °C)	P4_1_2_1_2

**Table 2:** A list of rationally selected ICCGY mutations, the applied strategy, and the potential interaction partner (if applicable). The introduced amino acid should interact with the potential partner listed, resulting in an electrostatic interaction. The “negative controls” should not induce any interaction. The ICCGY mutants were generated, and the in silico distances were calculated using PyMOL (v.2.3; [47]).

ICCGY Mutant	Applied Strategy: Electrostatic Interaction (EIA), Negative Control (NC)	Potential Interaction Partner; Distance (In Silico), Å
Q6E	EIA	R139; 3.3
T26E	NC	/
T110E	EIA	R119; 6.0
Q183K	NC	/
Q238K	NC	/

**Table 3:** An evaluation of μL batch crystallization experiments for the purified ICCGY wild type (WT) and mutants T26E, T110E, Q183K, and Q238K. Mutants Q6E and Q238K did not crystallize under the tested conditions within 56 h (/). The ICCGY variants were evaluated regarding crystallization start, crystal size, and amount (manually analyzed) at 1.2 M succinic acid (pH 7.0). Statistically relevant differences to ICCGY in crystal sizes are denoted as “*” (*p* < 0.01).

ICCGY Variant	Protein Concentration, g L^−1^	Crystallization Onset, h	Crystal Size, µm	Crystal Amount, -
WT	10	26	247.8 ± 70.9	25
	5	/	/	/
Q6E	/	/	/	/
T26E	10	22	344.5 ± 63.2 (*)	12
T110E	10	6	305.7 ± 78.2	21
	5	20	188.8 ± 84.7	16
Q183K	10	48	252.9 ± 97.2	12
Q238K	/	/	/	/

## Data Availability

All X-ray crystal structure data in this study were deposited and are available in the Protein Data Bank under the identification codes 9QYP (ICCG), 9QYU (ICCGY), 9QYQ (ICCGY T26E), 9QYT (ICCGY T110E), 9QYR (ICCGY Q183K), and 9QYS (ICCGY Q238K). All other data generated or analyzed during this study are included in this article and the Supplementary Materials or are available from the corresponding author upon reasonable request.

## Supplementary Materials

The following supporting information can be downloaded at https://www.mdpi.com/article/10.3390/bioengineering12060561/s1, Figure S1: An illustration of the crystal contact at position Y61 of (a) the ICCG structure (PDB ID: 6THT) used for the homology model and (b) in silico mutant Y61E; Figure S2: SDS-PAGE visualizing IMAC-purified ICCG WT and mutant L50Y (ICCGY) samples; Table S1: List of ICCG variants with partial overlapping forward (5′-3′) and reverse (3′-5′) oligonucleotides; Table S2: Data collection and refinement statistics of X-ray diffraction experiments of crystals from ICCG (short tag), ICCG L50Y (ICCGY), and ICCGY mutants T26E, T110E, Q183K, and Q238K.

## Abbreviations

The following abbreviations are used in this manuscript:

E (Glu)	Glutamic acid
EIA	Electrostatic interaction
HTP	High throughput
ICCG(Y)	LCC mutant: F243I/D238C/S283C/Y127G (L50Y)
IEX	Ion exchange chromatography
IMAC	Immobilized metal ion affinity chromatography
K (Lys)	Lysine
*Lb/Lk*ADH	*Lactobacillus brevis/kefiri* alcohol dehydrogenase
LCC	Leaf branch compost cutinase
NC	Negative control
*Nsp*ER1-L1,5	*Nostoc* sp. PCC 1720 ene reductase (with engineered Loop1,5)
PDB ID	Protein Data Bank Identification
PET	Poly(ethylene terephthalate)
Q (Gln)	Glutamine
R (Arg)	Arginine
RMSD	Root mean square deviation
SEC	Size exclusion chromatography
SDS-PAGE	Sodium dodecyl sulfate polyacrylamide gel electrophoresis
T (Thr)	Threonine
WT	Wild type
