# Supplementary material for: Application of a Rational Crystal Contact Engineering Strategy on a Poly(ethylene terephthalate)-Degrading Cutinase
Source: Bioengineering (Basel). 2025 May 23;12(6):561. doi: 10.3390/bioengineering12060561 (PMC12189717; doi:10.3390/bioengineering12060561)
Supplement: Supplementary file 1 [file bioengineering-12-00561-s001.zip › bioengineering-3632986-supplementary.pdf]

bioengineering

MDPI

Supplementary Material

Application of a rational crystal contact engineering strategy on a PET-degrading cutinase

Brigitte Walla [1], Anna-Maria Dietrich [1], Edwin Brames [1], Daniel Bischoff [1], Stefanie Fritzsche [2], Kathrin Castiglione [2], Robert Janowski [3], Dierk Niessing [3,4], and Dirk Weuster-Botz [1,*]

1 Chair of Biochemical Engineering, Technical University of Munich, Boltzmannstraße 15, 85748 Garching, Germany; brigitte.walla@tum.de (B.W.); daniel.bischoff@tum.de (D.B.)
2 Institute of Bioprocess Engineering, Department of Chemical and Biological Engineering, Friedrich-Alexander-Universität Erlangen-Nürnberg, Paul-Gordan-Straße 3, 91052 Erlangen, Germany
3 Molecular Targets and Therapeutics Center, Institute of Structural Biology, Helmholtz Zentrum München, Ingolstädter Landstraße 1, 85764 Neuherberg, Germany
4 Institute of Pharmaceutical Biotechnology, Ulm University, James-Franck-Ring N27, 89081 Ulm, Germany
* Correspondence: dirk.weuster-botz@tum.de (D.W.-B.)

Table S1. List of ICCG variants with partial overlapping forward (5′ - 3′) and reverse (3′ - 5′) oligonucleotides designed for QuikChange-PCR according to Zheng et al. [1]. Mutations are denoted in bold.

ICCG variant	5′ - 3′	3′ - 5′
L50Y	cacgtctt**tat**acgttcggtggcatc	cgaacgt**ata**agacgtgccggtcg
Q6E	cgtac**gaa**cgtggcccgaatcc	ccacg**ttc**gtacgggttgc
T26E	gtggca**gaa**tacaccgtctc3	cggtgta**ttc**tgccacgctaaac
T110E	ctgcgt**gaa**agttccccgag	gggaact**ttc**acgcaggtagttc
Q183K	ggtttcg**aaa**catgccatcc	ggcatg**ttt**cgaaaccggc
Q238K	cgttaccgc**aaa**ttcctgtgtaatg	aggaa**ttt**gcggtaacgggtgtc

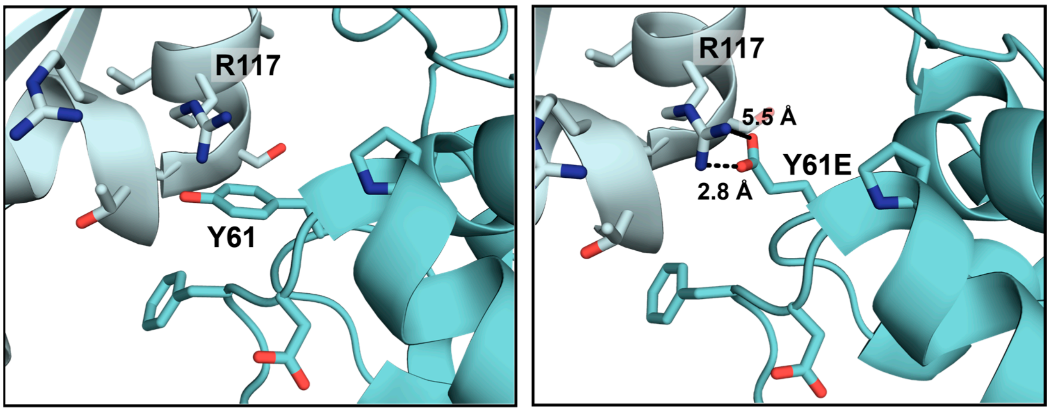

(**a**) (**b**)

Figure S1. Illustration of the crystal contact at position Y61 of (**a**) the ICCG structure (PDB ID: 6THT) used for the homology model, and (**b**) *in silico* mutant Y61E. The figures were generated and the *in silico* distance to the interaction partner R117 was calculated using PyMOL (v.2.3; [2]).

Table S2. Data collection and refinement statistics of X-ray diffraction experiments of crystals from ICCG (short tag), ICCG L50Y (ICCGY), and ICCGY mutants T26E, T110E, Q183K, and Q238K (values in parentheses are for highest resolution shell).

Variant	ICCG	ICCG L50Y (ICCGY)	ICCGY T26E	ICCGY T110E	ICCGY Q183K	ICCGY Q238K
**Data collection**						
**PDB ID**	9QYP	9QYU	9QYQ	9QYT	9QYR	9QYS
**Beamline**	PETRA III DESY BEAMLINE P11	PETRA III DESY BEAMLINE P11	PETRA III DESY BEAMLINE P11	PETRA III DESY BEAMLINE P11	PETRA III DESY BEAMLINE P11	PETRA III DESY BEAMLINE P11
**Wavelength, Å**	1.00	1.00	1.00	1.00	1.00	1.00
**Space group**	I 2 2 2	P 41 21 2	P 41 21 2	P 41 21 2	P 41 21 2	P 41 21 2
**Cell constants a b c, Å α β γ, deg**	97.91 101.87 265.81 90.00 90.00 90.00	96.65 96.65 73.71 90.00 90.00 90.00	97.12 97.12 73.93 90.00 90.00 90.00	97.21 97.21 74.67 90.00 90.00 90.00	96.57 96.57 73.16 90.00 90.00 90.00	97.32 97.32 72.48 90.00 90.00 90.00
**Chains per asymmetric unit**	3	1	1	1	1	1
**Resolution, Å**	48.95-2.01 (2.13-2.01)	48.33-1.51 (1.60-1.51)	48.56-1.70 (1.80-1.70)	48.60-1.64 (1.74-1.64)	48.28-1.71 (1.81-1.71)	48.66-1.80 (1.91-1.80)
**CC (1/2)**	1.00 (0.50)	1.00 (0.68)	1.00 (0.54)	0.99 (0.52)	1.00 (0.81)	1.00 (0.77)
**Refinement**						
**Resolution, Å**	2.01	1.51	1.70	1.64	1.71	1.80
**R_{work} / R_{free}**	0.21 / 0.25	0.15 / 0.18	0.18 / 0.21	0.20 / 0.23	0.17 / 0.20	0.175 / 0.213
**No. atoms**						
**Protein**	5870	1971	1982	1982	1989	1971
**Water**	332	205	227	203	292	206
**Other**	0	25	2	9	2	4
**B-factors**						
**Overall**	45.64	31.78	32.96	32.47	28.78	36.16
**Protein main chain**	44.56	30.22	31.69	31.34	27.48	35.06
**Protein side chain**	46.20	32.79	33.68	33.10	29.53	36.73
**R.m.s. deviations**						
**Bond lengths, Å**	0.01	0.01	0.01	0.01	0.01	0.01
**Bond angles, °**	1.91	1.59	1.93	1.93	1.98	1.93
**Ramachandran plot**						
**Most favored, %**	92.03	94.94	94.94	94.16	94.55	94.94
**Additional allowed, %**	5.91	3.11	3.11	4.28	3.89	2.72

Comparing the protein production and processing of ICCG and ICCGY, a difference in the heterogeneity of IMAC-purified samples, separated by SDS-PAGE (Figure S2), was visible. ICCGY tends to focus more on the height of the monomer (28 kDa), whereas ICCG shows an increased intensity for a band at ~48 kDa compared to ICCGY.

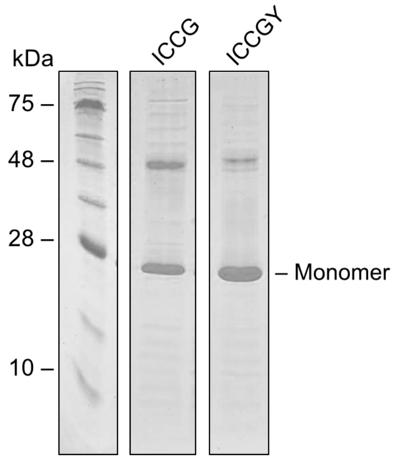

Figure S2. SDS-PAGE visualizing IMAC-purified ICCG WT and mutant L50Y (ICCGY) samples (0.5 g L^{-1}, 300 V, 35 mA, 50 min). Intensely focused bands are detected at ~28 kDa, corresponding to its molecular monomer weight of 28.83 kDa (calculated with ExPASy ProtParam) and between 48-63 kDa. Marker: BlueStar prestained protein marker (Nippon Genetics Europe GmbH).

References

1. Zheng, L., Baumann, U., and Reymond, J. L. "An efficient one-step site-directed and site-saturation mutagenesis protocol." *Nucleic Acids Res* 32, no. 14 (2004): e115. 10.1093/nar/gnh110
2. Schrödinger, L., & DeLano, W. : Pymol, Version 2.3 (2020). Retrieved from http://www.pymol.org/pymol
